# Early identification of severe community-acquired pneumonia: a retrospective observational study

**DOI:** 10.1136/bmjresp-2019-000438

**Published:** 2019-06-05

**Authors:** Frances S Grudzinska, Kerrie Aldridge, Sian Hughes, Peter Nightingale, Dhruv Parekh, Mansoor Bangash, Rachel Dancer, Jaimin Patel, Elizabeth Sapey, David R Thickett, Davinder P Dosanjh

**Affiliations:** 1 Institute of Inflammation and Ageing, University of Birmingham College of Medical and Dental Sciences, Birmingham, UK; 2 Queen Elizabeth Hospital Birmingham, Birmingham, UK; 3 Institute of Inflammation and Ageing, University of Birmingham, Birmingham, UK

**Keywords:** community acquired pneumonia, CAP, sepsis, CURB65, qsofa, lac-curb-65, NEWS

## Abstract

**Background:**

Community-acquired pneumonia (CAP) is a leading cause of sepsis worldwide. Prompt identification of those at high risk of adverse outcomes improves survival by enabling early escalation of care. There are multiple severity assessment tools recommended for risk stratification; however, there is no consensus as to which tool should be used for those with CAP. We sought to assess whether pneumonia-specific, generic sepsis or early warning scores were most accurate at predicting adverse outcomes.

**Methods:**

We performed a retrospective analysis of all cases of CAP admitted to a large, adult tertiary hospital in the UK between October 2014 and January 2016. All cases of CAP were eligible for inclusion and were reviewed by a senior respiratory physician to confirm the diagnosis. The association between the CURB65, Lac-CURB-65, quick Sequential (Sepsis-related) Organ Failure Assessment tool (qSOFA) score and National Early Warning Score (NEWS) at the time of admission and outcome measures including intensive care admission, length of hospital stay, in-hospital, 30-day, 90-day and 365-day all-cause mortality was assessed.

**Results:**

1545 cases were included with 30-day mortality of 19%. Increasing score was significantly associated with increased risk of poor outcomes for all four tools. Overall accuracy assessed by receiver operating characteristic curve analysis was significantly greater for the CURB65 and Lac-CURB-65 scores than qSOFA. At admission, a CURB65 ≥2, Lac-CURB-65 ≥moderate, qSOFA ≥2 and NEWS ≥medium identified 85.0%, 96.4%, 40.3% and 79.0% of those who died within 30 days, respectively. A Lac-CURB-65 ≥moderate had the highest negative predictive value: 95.6%.

**Conclusion:**

All four scoring systems can stratify according to increasing risk in CAP; however, when a confident diagnosis of pneumonia can be made, these data support the use of pneumonia-specific tools rather than generic sepsis or early warning scores.

**Key messages**

**What is the key question?**

- What risk stratification tool should you use in community-acquired pneumonia?

**What is the bottom line?**

- Pneumonia-specific tools provide better discrimination of patients at high risk of adverse outcome than generic sepsis tools.

**Why read on?**

- This paper assesses commonly used risk stratification tools in a pragmatic patient population comparing newer tools such as quick Sequential (Sepsis-related) Organ Failure Assessment tool with established scores.

## Introduction

Community-acquired pneumonia (CAP) is the fourth leading cause of death worldwide when combined with lower respiratory tract infections.^1^ It is associated with significant mortality^2^ and frequently leads to sepsis^3 4^ with mortality rates rising to 30%.^5^ Early identification of patients with severe CAP enables modification of management strategies and improves outcomes for patients.^6–8^

To identify those at risk of poor outcomes, guidelines for management of CAP and sepsis suggest risk stratification tools should be used^9–11^; however there is no consensus as to which tool should be used.^11–16^

Severity assessment tools have been developed specifically for identifying patients at risk of deterioration due to sepsis. The quick Sequential (Sepsis-related) Organ Failure Assessment tool (qSOFA)^15^ is the recommended tool to screen patients with suspected infection outside the intensive care unit (ICU)^11 17–19^ (one point for each of altered mentation, respiratory rate (RR) ≥22 and systolic blood pressure (SBP) ≤100 mm Hg, with a score ≥2 suggesting high risk for deterioration).^15^ More generic tools designed to predict deterioration regardless of aetiology have also been designed, such as the National Early Warning Score (NEWS), widely used in the English National Health Service.^16^ NEWS is a composite score assessing level of alertness, RR, blood pressure (BP), heart rate, oxygen saturation and temperature with increasing values for more abnormal measurements (see online supplementary eTable 1 for a full description). A score of ≥3 in any category or score ≥5 overall triggers urgent patient review.

10.1136/bmjresp-2019-000438.supp1 Supplementary data

Disease-specific tools, such as CURB65, are recommended by respiratory societies worldwide.^9 10 20^ Each of altered mentation, blood urea >7.0, RR ≥30, SBP <90 or diastolic BP ≤60 and age ≥65 scores one point, with scores ≥2 considered moderate–severe. Original validation of this tool, however, excluded patients from long-term care facilities as well as those with common comorbidities.^12^

In addition, attempts have been made to refine previously well-described scores by using biomarkers such as lactate. Lactate is a strong independent predictor of mortality in both pneumonia and sepsis,^13 21^ and work by other groups has shown that addition of lactate ≥1.8 mmol/L improves the ability of CURB65 to predict mortality.^7 13^

Existing evidence supports early intervention and consideration of ICU for appropriate patients^8 12 22^ using severity assessment tools to aid decision-making; however, the evidence to support one tool over another is lacking in patients with pneumonia. We compared the performance of four commonly used severity assessment tools (CURB65, Lac-CURB-65, NEWS and qSOFA) in a CAP-specific population to identify those at risk of adverse outcomes. We selected these four scores as they are commonly used in clinical practice and most widely recommended by sepsis and respiratory societies. We hypothesise that pneumonia-specific tools will more accurately predict patients at high risk of adverse outcomes.

## Methods

### Study institution and subjects

All adults admitted to the Queen Elizabeth Hospital Birmingham, UK with CAP between October 2014 and January 2016 were eligible for inclusion.

CAP cases were identified using the hospital coding system retrospectively. CAP was defined using British Thoracic Society (BTS) guidelines.^9^ Senior respiratory physicians confirmed the diagnosis of CAP using admission documents, radiology and electronic patient records. Cases were excluded if there were no new infiltrates in relevant radiological investigations. We identified patients who would have been previously identified as healthcare-associated pneumonia (HCAP). Patients with hospital-acquired pneumonia (HAP) were excluded. HAP and HCAP were defined using the 2005 American Thoracic Society (ATS) and Infectious Diseases Society of America guidelines.^10^ Ethics was deemed not to be required based on the Health Research Authority decision tool.^23^ This was confirmed by our institution’s research team and local approval was granted.

In addition to the first set of physiological observations recorded on admission to hospital (level of alertness, respiratory rate, temperature, oxygen saturations, BP and heart rate), the first set of biochemical and haematological laboratory results were also collected from the electronic patient record.

CURB65,^12^ Lac-CURB-65,^13^ NEWS^16^ and qSOFA^15^ scores were calculated as previously described.

To assess for confusion in qSOFA, CURB65 and Lac-CURB-65, we reviewed the admission document and scored for confusion if any of the following were documented: abnormal AVPU score (alert, response to voice, pain or unresponsive), Glasgow coma scale ≤13, abnormal mental state examination, or documentation of confusion or delirium.

Lac-CURB-65 score and NEWS were grouped into predefined ‘Low’, ‘Moderate’ and ‘High’ risk categories. Lac-CURB-65 cut-offs: low—CURB-65 ≤1 and/or lactate <2.0 mmol/L; moderate—CURB65=2 and/or lactate 2.0–4.0 mmol/L; high—CURB65 ≥3 and/or lactate >4.0 mmol/L.^13^

NEWS cut-offs: low—aggregate score 1–4; medium—aggregate score 5–6 or a score of ≥3 in a single category; high—aggregate score ≥7 as previously defined.^16^

Outcome measures included admission to ICU, length of stay and in-hospital, 30-day, 90-day and 365-day mortality; data were collected from the electronic patient record.

### Patient and public involvement

Patient and the public were not involved in the development of this research. This study was undertaken in accordance with the Strengthening the Reporting of Observational Studies in Epidemiology guidelines for cohort studies.

### Statistical analysis

Comparison of proportions was performed using the χ^2^ test for trend; trends in median length of stay were assessed using the Jonckheere-Terpstra test. Sensitivity, specificity, receiver operating characteristic (ROC) curve analysis, positive and negative predictive values, and likelihood ratios were calculated for each scoring system. Cases with missing data were excluded from analysis on a score-by-score basis. To assess the effect of missing data, analyses were repeated using multiple imputation and assumption of normal values where data points were absent. These analyses and detailed explanation of methods are presented in the online supplement. Statistical analysis was carried out using IBM SPSS Statistics for Windows V.24.0 and R (V.3.4.4, Vienna, Austria).

## Results

### Participant demographics

**Figure 1:**
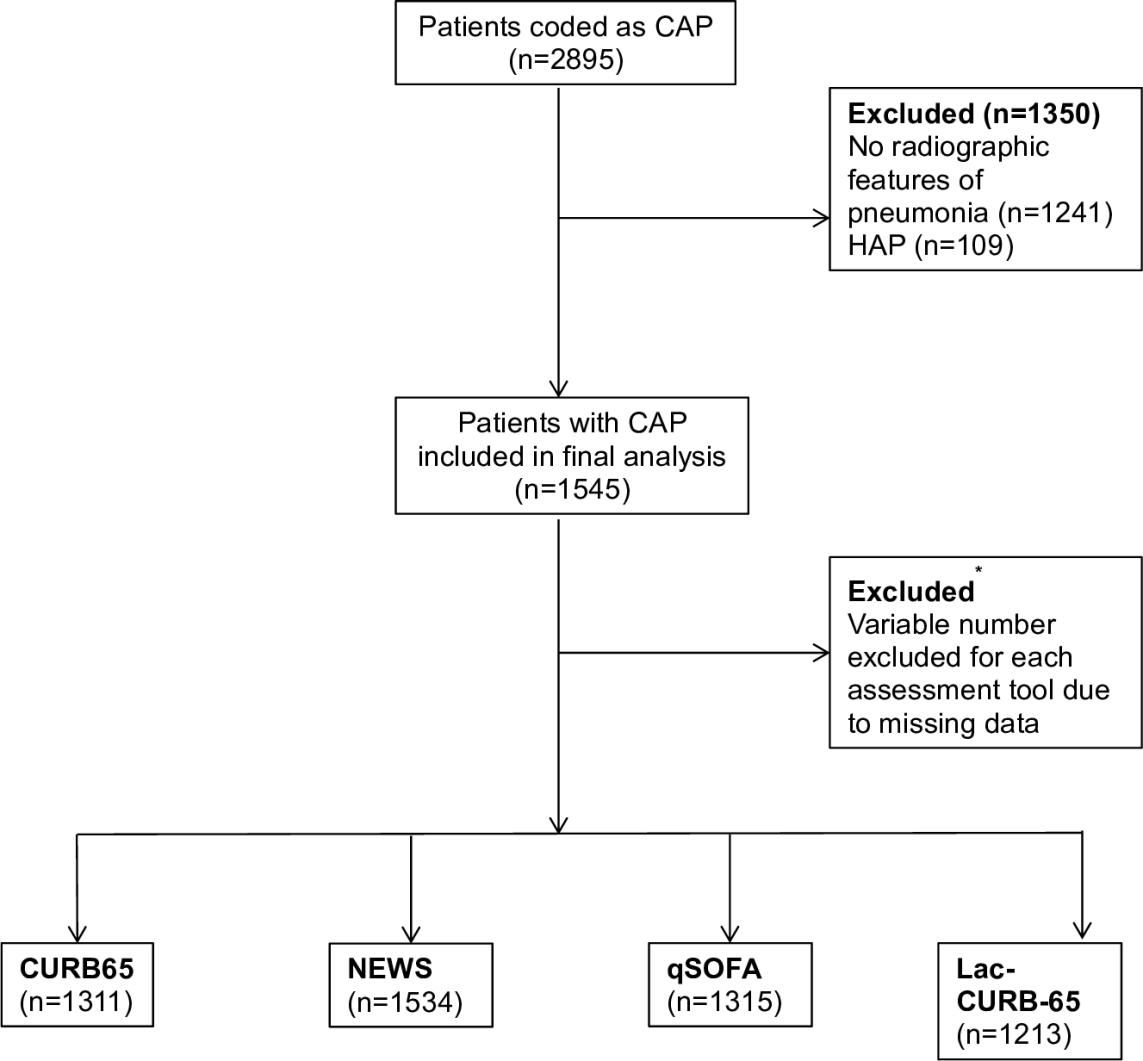
Modified CONSORT diagram demonstrating patient inclusion and exclusion pathways. *Reasons for exclusion of patients for each severity assessment tool (number of cases excluded). Some cases were excluded due to more than one missing data point: CURB65: Confusion (230), urea (5), respiratory rate (4), blood pressure (4), age >65 years (0). Lac-CURB-65: as for CURB65 plus lactate (227). qSOFA: mentation (230), respiratory rate (4), blood pressure (4). NEWS: temperature (9), oxygen saturations (5), level of consciousness (4), respiratory rate (4), blood pressure (4), heart rate (4). CAP, community-acquired pneumonia; HAP, hospital-acquired pneumonia; NEWS, National Early Warning Score; qSOFA, quick Sequential (Sepsis-related) Organ Failure Assessment.

A total of 2895 patients were coded as having CAP and 1545 were included in the final analysis (figure 1). Due to missing data, there were variable numbers of cases included in the analysis for each score (figure 1). For a detailed comparison of missing and included cases, see the online supplementary eTables 3–7.

The median age of patients included was 76 (IQR 63–85). Of all cases, 50.8% (785) were men; 29.0% (449) of cases fulfilled the criteria for what was previously defined as HCAP. Eighty-nine per cent of cases had at least one comorbidity (online supplementary eTable 2). Overall 30-day mortality was 19.0%; in-hospital mortality was 15.4% with an ICU admission rate of 6.4%. Full demographic and outcome information is available in the online supplement (online supplementary eTable 2).

### Validation of CURB65 for patients previously defined as HCAP

In 2005, HCAP was defined as a separate entity to CAP in order to describe a population of patients in long-term care or receiving home-based or hospital-based intravenous therapy or dialysis who had increased mortality^24^ and high prevalence of antibiotic-resistant pathogens.^25^ The concept of HCAP has more recently been rejected; however, the original validation of the CURB65 score excluded those that were labelled as HCAP. This has led to widespread use of CURB65 in a patient population it was not originally validated in. We analysed the non-HCAP and HCAP groups separately for CURB65 to ensure that there was no significant difference in risk stratification between the two groups.

CURB65 scoring was possible for 1311 (84.9%) of all cases, with complete data available for 83.5% (375) of patients with HCAP and 85.4% (936) of patients without HCAP (table 1).

**Table 1:** CURB65 as a prognostic tool for different outcome measures stratified by CAP aetiology

Outcome	CURB65 score						P value
	0	1	2	3	4	5	
n							
All	173	287	395	309	129	18	
HCAP	27	65	106	113	54	10	
Non-HCAP	146	222	289	196	75	8	
30-day mortality n (%)							
All	6 (3.5)	33 (11.5)	73 (18.5)	83 (26.9)	58 (45.0)	7 (38.9)	<0.001
HCAP	0 (0.0)	10 (15.4)	25 (23.6)	36 (31.9)	27 (50.0)	3 (30.0)	<0.001
Non-HCAP	6 (4.1)	23 (10.3)	48 (16.6)	47 (24.0)	31 (41.3)	4 (50.0)	<0.001
90-day mortality n (%)							
All	10 (5.8)	45 (15.7)	103 (26.1)	108 (35.0)	65 (50.4)	7 (38.9)	<0.001
HCAP	1 (3.7)	15 (41.5)	28 (39.6)	46 (47.8)	29 (63.0)	3 (30.0)	<0.001
Non-HCAP	9 (6.2)	30 (13.5)	75 (26.0)	62 (31.6)	36 (48.0)	4 (50.0)	<0.001
365-day mortality n (%)							
All	11 (6.4)	69 (24.0)	132 (33.4)	143 (46.3)	73 (56.6)	7 (38.9)	<0.001
HCAP	1 (3.7)	27 (41.5)	42 (39.6)	54 (47.8)	34 (63.0)	3 (30.0)	<0.001
Non-HCAP	10 (6.9)	42 (18.9)	90 (31.1)	89 (45.4)	39 (52.0)	4 (50.0)	<0.001
In-hospital death n (%)							
All	3 (1.7)	25 (8.7)	60 (15.2)	67 (21.7)	48 (37.2)	5 (27.8)	<0.001
HCAP	0 (0.0)	8 (12.3)	17 (16.0)	25 (22.1)	19 (35.2)	1 (10.0)	<0.001
Non-HCAP	3 (2.1)	17 (7.7)	43 (14.9)	42 (21.4)	29 (38.7)	4 (50.0)	<0.001
ICU admission n (%)							
All	12 (6.9)	15 (5.2)	39 (9.9)	13 (4.2)	6 (4.7)	2 (11.1)	0.514
HCAP	2 (7.4)	3 (4.6)	4 (3.8)	2 (1.8)	1 (1.9)	1 (10.0)	0.285
Non-HCAP	10 (6.8)	12 (5.4)	35 (12.1)	11 (5.6)	5 (6.7)	1 (12.5)	0.733
Length of inpatient stay median days (IQR)							
All	3 (1.0–7.0)	6 (3.0–13.0)	8 (4.0–15.0)	9 (5.0–17.0)	8 (4.0–13.5)	8.5 (4.5–14.0)	<0.001
HCAP	5 (2.0–10.0)	9 (4.0–17.5)	9 (5.0–16.3)	8 (4.5–15.5)	8 (5.0–12.0)	7.5 (4.5–17.3)	0.529
Non-HCAP	3 (1.0–7.0)	6 (3.0–11.0)	8 (4.0–14.5)	9.5 (5.0–20.8)	7 (4.0–16.0)	9 (3.8–12.3)	<0.001

Comparison of proportions performed using χ^2^ test for trend. Trends in median length of stay assessed using the Jonckheere-Terpstra test.

CAP, community-acquired pneumonia; HCAP, healthcare-associated pneumonia;ICU, intensive care unit.

CURB65 score was able to stratify according to increasing risk of in-hospital mortality as well as 30-day, 90-day and 365-day mortality (table 1). Increasing CURB65 score was not associated with increased likelihood of ICU admission (table 1). Increasing length of stay was significantly associated with increased CURB65 score in the whole cohort as well as the non-HCAP group, but not in the HCAP-alone group.

### Ability of different severity scoring systems to risk stratify

Increasing NEWS and qSOFA scores were significantly associated with increased risk of ICU admission during admission (table 2). Increasing severity score was significantly associated with increased risk of mortality for all four scoring systems. Increasing scores were also associated with increased length of stay for all scoring tools.

**Table 2:** Ability of severity assessment tools to risk stratify for outcome measures in CAP

Outcome	CURB65						P value
	0 n=173	1 n=287	2 n=395	3 n=309	4 n=129	5 n=18	
30-day mortality n (%)	6 (3.5)	33 (11.5)	73 18.5)	83 (26.9)	58 (45.0)	7 (38.9)	<0.001
90-day mortality n (%)	10 (5.8)	45 (15.7)	103 (26.1)	108 (35.0)	65 (50.4)	7 (38.9)	<0.001
365-day mortality n (%)	11 (6.4)	69 (24.0)	132 (33.4)	143 (46.3)	73 (56.6)	7 (38.9)	<0.001
In-hospital death n (%)	3 (1.7)	25 (8.7)	60 (15.2)	67 (21.7)	48 (37.2)	5 (27.8)	<0.001
ICU admission n (%)	12 (6.9)	15 (5.2)	39 (9.9)	13 (4.2)	6 (4.7)	2 (11.1)	0.514
Length of inpatient stay median days (IQR)	3 (1.0–7.0)	6 (3.0–13.0)	8 (4.0–15.0)	9 (5.0–17.0)	8 (4.5–13.5)	9 (4.5–14.0)	<0.001
	**Lac-CURB-65**						
** **	**Low** **n** **=** **204**		**Moderate** **n** **=** **506**		**High** **n** **=** **503**		
30-day mortality n (%)	9 (4.4)		77 (15.2)		166 (33.0)		<0.001
90-day mortality n (%)	15 (7.4)		107 (26.8)		199 (39.6)		<0.001
365-day mortality n (%)	29 (14.2)		143 (28.3)		243 (48.3)		<0.001
In-hospital death n (%)	5 (2.5)		62 (12.3)		134 (26.6)		<0.001
ICU admission n (%)	10 (4.9)		41 (8.1)		36 (7.1)		0.483
Length of inpatient stay median days (IQR)	6 (2.0–10.0)		7 (4.0–13.0)		8 (5.0–16.0)		<0.001
	**NEWS**						
	**Low** **n** **=** **557**		**Moderate** **n** **=** **417**		**High** **n** **=** **560**		
30-day mortality n (%)	61 (11.0)		61 (14.6)		168 (30.0)		<0.001
90-day mortality n (%)	96 (17.2)		93 (22.3)		197 (35.2)		<0.001
365-day mortality n (%)	137 (24.6)		125 (30.0)		243 (43.4)		<0.001
In-hospital death n (%)	56 (10.1)		52 (12.5)		127 (22.7)		<0.001
ICU admission n (%)	12 (2.1)		25 (6.0)		58 (10.4)		<0.001
Length of inpatient stay median days (IQR)	6 (3.0–12.0)		7 (4.0–16.0)		8 (4.0–14.8)		<0.001
	**qSOFA score**						
	**0** **n=** **369**		**1** **n=** **629**	**2** **n=** **265**	**3** **n=** **52**		
30-day mortality n (%)	43 (11.7)		114 (18.4)	82 (30.9)	24 (46.2)		<0.001
90-day mortality n (%)	62 (16.8)		154 (24.5)	100 (37.7)	25 (48.1)		<0.001
365-day mortality n (%)	86 (23.3)		205 (32.6)	119 (44.9)	28 (53.8)		<0.001
In-hospital death n (%)	36 (9.8)		86 (13.7)	68 (25.7)	20 (38.5)		<0.001
ICU admission n (%)	13 (3.5)		49 (7.8)	22 (8.3)	3 (5.8)		0.038
Length of inpatient stay median days (IQR)	6 (3.0–11.0)		7 (4.0–15.0)	8 (4.0–15.0)	8 (5.0–15.75)		<0.001

Comparison of proportions done using χ^2^ test for trend. Trends in median length of stay assessed using the Jonckheere-Terpstra test.

Lac-CURB-65 cut-offs: low—CURB-65 ≤1 and/or lactate <2.0 mmol/L; moderate—CURB65=2 and/or lactate 2.0–4.0 mmol/L; high—CURB65 ≥3 and/or lactate >4.0 mmol/L. NEWS score cut-offs: low—aggregate score 1–4; medium—aggregate score 5–6 or a score of ≥3 in a single category; high—aggregate score ≥7 as previously defined.^13 16^

CAP, community-acquired pneumonia; ICU, intensive care unit; NEWS, National Early Warning Score; qSOFA, quick Sequential (Sepsis-related) Organ Failure Assessment.

### Overall accuracy of the different scoring systems to predict 30-day mortality

**Figure 2:**
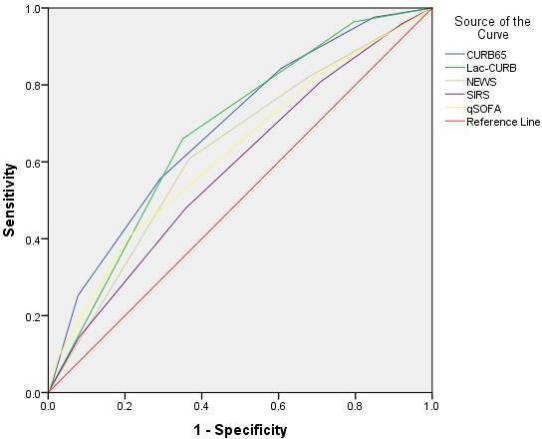
Receiver operating characteristic (ROC) curves to assess overall accuracy of severity assessment tools using 30-day mortality as the standard. Area under the ROC curve for CURB65, Lac-CURB-65, NEWS and qSOFA were 0.69, 0.68, 0.63 and 0.62, respectively. NEWS, National Early Warning Score; qSOFA, quick Sequential (Sepsis-related) Organ Failure Assessment.

Overall accuracy of the scoring systems to identify those at risk of death within 30 days of presentation to hospital was calculated using ROC curve analysis (figure 2). The area under the ROC curve (AUROC) for CURB65, Lac-CURB-65, NEWS and qSOFA were 0.69, 0.68, 0.63 and 0.62, respectively. AUROC values were significantly greater for CURB65 and Lac-CURB-65 scoring systems when compared with those generated using the qSOFA criteria (CURB65 vs qSOFA p<0.0001, Lac-CURB-65 vs qSOFA p=0.0024) (table 3).

**Table 3:** Comparison of overall accuracy of severity assessment tools to predict 30-day mortality at admission

(P value)	CURB65	Lac-CURB-65	NEWS	qSOFA
CURB65				
Lac-CURB-65	0.4827			
NEWS	0.0054	*0.0138*		
qSOFA	*<0.001*	*0.0024*	0.7858	

Receiver operating curve analysis was performed and the table presents the p values following comparison of the area under the curve for each assessment tool using 30-day mortality as the standard. Comparison was performed using DeLong’s test.

NEWS, National Early Warning Score; qSOFA, quick Sequential (Sepsis-related) Organ Failure Assessment.

### Performance characteristics of severity assessment tools

With 30-day mortality as the outcome measure, we calculated the performance characteristics of each of the scoring systems using previously defined cut-off points.^13 15^ Lac-CURB-65, using ‘moderate’ as the cut-off, had the greatest sensitivity and negative predictive value (NPV), 96.4% and 95.6%, respectively. This was closely followed by CURB65 with a cut-off of ≥2 giving a sensitivity of 85.0% and NPV of 91.5%. qSOFA had the poorest sensitivity at 40.3%, but relatively high specificity of 79.9% (table 4).

**Table 4:** Performance characteristics of the severity scoring systems using 30-day mortality as the outcome measure

Score (n)	Sensitivity (%)	Specificity (%)	PPV (%)	NPV (%)	NLR	PLR
CURB65 ≥2 (851)	85.0	40.1	26.0	91.5	0.37	1.42
CURB65 ≥3 (456)	56.9	70.1	32.5	86.9	0.61	1.94
Lac-CURB-65 ≥moderate (1009)	96.4	20.3	24.1	95.6	0.18	1.21
Lac-CURB-65 high (503)	65.9	64.9	33	87.9	0.53	1.88
qSOFA* (317)	40.3	79.9	33.4	84.3	0.75	2.01
NEWS ≥medium (997)	79	39.9	23.4	89.1	0.53	1.31
NEWS high (560)	57.9	68.5	30.0	87.5	0.61	1.84

*Cut-off value for qSOFA was ≥2.

NEWS, National Early Warning Score; NLR, negative likelihood ratio;NPV, negative predictive value;PLR, positive likelihood ratio;PPV, positive predictive value;qSOFA, quick Sequential (Sepsis-related) Organ Failure Assessment.

### Assessment of the impact of missing values on the analysis

To assess the impact of missing data, patient characteristics and outcome measure data were compared between those with complete data and those without for each severity assessment tool (online supplementary eTables 3–7). The complete analysis was repeated having replaced the absent data with either normal values or by using multiple imputation. The full results of these analyses can be reviewed in the online supplement (see online supplementary eTables 8–14). Both the assumed normal and multiple imputation analyses resulted in little significant change in the results.

## Discussion

This study describes a large cohort of hospitalised CAP and confirms that CURB65, Lac-CURB-65, NEWS and qSOFA scores at the time of hospital admission can stratify according to increasing risk of mortality in all patients with CAP. These data also suggest that using a ‘moderate’ Lac-CURB-65 score as a threshold for identifying those at increased risk of 30-day mortality may have utility as a ‘rule-out’ when assessing patients that may need escalation of care.

A key strength of this study was the use of a pragmatic approach to patient inclusion, which has led to the validation of these assessment tools in patients often excluded from other studies but among which the severity assessment scores are commonly used. Patients excluded from the original validation of the CURB65 score included those with bronchiectasis, malignancy, prior hospital admission within 14 days, immunocompromise, nursing home residents or where pneumonia was an expected terminal event.^12^ The generalisability of our findings to real-life patient populations has been increased by including these patients.

A previous study has demonstrated that CURB65 had greater predictive ability for adverse outcomes in CAP than systemic inflammatory response syndrome criteria or early warning scores^26^; however, we have used additional, comparatively novel scoring systems and applied them to a larger cohort of patients with more pragmatic inclusion criteria and measured long-term mortality outcomes.^26^

The diagnosis of pneumonia has been verified by the review of radiological and clinical findings. A key finding of the UK-wide BTS pneumonia audit was that using clinical coding alone led to misdiagnosis in approximately a third of cases due to lack of clinicoradiographic features of pneumonia,^27^ a finding borne out by this study.

Increasing NEWS and qSOFA scores were associated with increased rate of admission to ICU. It should be noted that during the study period, all scores were being used in clinical practice, except for qSOFA, and this may have had an impact on the decision-making process when a patient was admitted to ICU. Our ICU admission rate is lower than that seen in studies performed outside the UK^7 15 28^; however, it is in keeping with the BTS pneumonia audit.^27^ This is likely to be due to inclusion of patients with treatment limitations; we choose to include patients with treatment limitations to enable application of these scores to all patients admitted. Prediction of adverse outcome remains important for all patients, even if they would be unlikely to benefit from ICU admission as it informs decision-making regarding appropriate interventions that can be implemented, as well as informing decisions regarding withdrawal of care in cases where further treatment may be futile.

The qSOFA tool was designed as a quick and easy screening tool, to allow repeated and widespread use to identify deteriorating patients.^15^ It was interesting to note that the sensitivity of the qSOFA score to predict 30-day mortality, when performed at the time of admission, was low in this CAP population, an observation that has been made in previous studies.^28 29^ qSOFA was more accurate at predicting ICU admission in this study and previous work.^30^ This suggests that though serial scoring may have use in identifying those that are deteriorating, in this cohort of patients with CAP, there was little use of the score as an indicator of 30-day mortality at the time of admission. The validation study for qSOFA defined adverse outcome as in-hospital mortality or ICU admission for greater than 3 days^15^; our different definition of adverse outcome may also affect interpretation of these data.

A raised lactate has been consistently demonstrated to be an independent predictor of mortality in sepsis^21^ and pneumonia.^7^ Frenzen *et al* found that addition of lactate ≥1.8 mmol/L significantly improved the ability of CURB65 to predict a combined endpoint of ICU admission and inpatient mortality,^7^ similarly confirmed by Chen and Li.^13^ However, this effect was not observed in our cohort for ICU admission or 30-day mortality. This is likely to be due to key differences in study design and populations. For example, Frenzen *et al* excluded any patients with treatment limitations and had a high ICU admission rate (22%) with very low mortality (7%). Our mortality rates were in keeping with those from the BTS pneumonia audit^27^ and large European cohorts.^2^ Thirty-day mortality was higher than in-hospital mortality, and this is likely to reflect the increased long-term mortality^31^ and high rates of re-admission seen after CAP^27^; this is especially true for older people as seen in this study.

Increasing age is well recognised to be an independent risk factor for mortality in CAP^32^ and is represented in CURB65. Greater than two-thirds of participants in this cohort were ≥65 years of age, meaning they score highly when using CURB65, whereas NEWS and qSOFA do not account for age. In the future, it would be pertinent to assess for impact of age on scoring systems to see if dichotomising by age criteria improves predictive ability.

To compare the overall accuracy of the scores to predict 30-day mortality, ROC curves were calculated. Though CURB65 and Lac-CURB-65 resulted in a significantly greater AUROC compared with qSOFA, the clinical significance of this difference is difficult to define, and none of the scores provided excellent discrimination of patients at high risk of adverse outcomes. The use of different inclusion criteria and management strategies, combined with different outcome measures used in previous studies, makes direct comparison with our findings challenging. The AUROC for the CURB65 has been reported as ranging from 0.71^7^ to 0.829^8 28 33^ in CAP populations (with patients with HCAP excluded), to 0.65^34^ in a cohort which included patients with HCAP, similar to findings presented here. The use of CURB65 in the HCAP population has been validated previously by Ewig *et al*.^35^

Goulden *et al* 18 used NEWS and qSOFA to predict mortality in a group of emergency admissions with sepsis and also found similar AUROCs to those presented here (0.65 and 0.62, respectively). A meta-analysis of qSOFA in predicting mortality identified an AUROC of 0.67; however, the sensitivity of qSOFA was very low.^29^ Brabrand and Henriksen found that CURB65 was not superior to NEWS in predicting 30-day mortality.^36^ A large CAP cohort study using the CAPNETZ^37^ database found that qSOFA plus age ≥65 years was as good at predicting 30-day mortality as CRB65.^38^ Data presented here for patients with CAP support other data in the literature and may suggest that the qSOFA score may not perform as well in a CAP-specific population when compared with a mixed sepsis population. The low AUROCs seen for these scores and by other groups demonstrate the weaknesses of these severity scoring systems in common clinical practice and highlight the need for better tools.

This study has limitations, including the retrospective single-centre study design and missing data. The most common missing data was documentation of the patient’s mental state. This may have introduced bias when comparing the different scoring systems. To account for this, we have presented analyses using multiple imputation and assumption of normal values. It is reassuring to note that there were no significant changes in the results when these alternative analysis methods were employed. With regards to prediction of ICU admission, we did not exclude patients with treatment limitations and this may have impacted on accuracy of these scores to predict ICU admission. Prospective multicentre studies to ensure collection of complete data sets and ensure generalisability are needed. In addition, further studies are warranted to examine the role of serial scoring to predict deterioration during an admission, rather than assessing risk at the time of admission. NEWS and qSOFA have already demonstrated validity for serial scoring^16 17^; however, this has not been assessed for the pneumonia-specific scores.

We recognise that other severity scoring tools exist for pneumonia and are more widely used outside the UK^39^; however, we opted for commonly used and simple scores that could be calculated at the point of admission rather than complex tools such as pneumonia severity index.

None of the commonly used existing tools provide excellent discrimination of patients at high risk of adverse outcomes, and more sophisticated scoring systems exist such as SOFA for sepsis or ATS minor criteria which provide better discrimination. However, refinement of existing simple tools or investigation of novel markers for poor prognosis in CAP would be beneficial. Furthermore, these data do not assist with the risk stratification of patients with HAP, and further studies are needed in this patient population. The development of an accurate risk stratification tool for CAP and HAP could lead to earlier identification of patients who would benefit from early escalation and targeted treatment.^12 22^

## Conclusion

These data suggest that four commonly used severity assessment tools are able to stratify patients according to increasing risk of mortality. Furthermore, a ‘low’ Lac-CURB-65 score appears to indicate that a poor outcome is unlikely. Tools specifically designed for sepsis and early recognition of patients at increased risk of ICU admission did not perform as well as the CAP-specific tools, particularly when compared with previous studies of all-cause sepsis, suggesting that organ-specific severity assessment tools may have greater use in early recognition of patients who are at risk of adverse outcomes.
