# Supplementary material for: Early identification of severe community-acquired pneumonia: a retrospective observational study
Source: BMJ Open Respir Res. 2019 Jun 5;6(1):e000438. doi: 10.1136/bmjresp-2019-000438 (PMC6561385; doi:10.1136/bmjresp-2019-000438)
Supplement: Supplementary data [file bmjresp-2019-000438supp001.pdf]

Online Data Supplement: Early identification of those with severe community acquired pneumonia: a retrospective observational study

Frances S. Grudzinska, Kerrie Aldridge, Sian Hughes, Peter Nightingale, Dhruv Parekh, Mansoor Bangash, Rachel CA. Dancer, Jaimin Patel, Elizabeth Sapey, David R.Thickett, Davinder PS. Dosanjh

Contents

Online Data Supplement: Early identification of those with severe community acquired pneumonia: a retrospective observational study 1

eTable 1-Scoring Parameters for NEWS score 3

eTable 2-Demographics, characteristics and outcomes of study cohort 4

Comparison of included and excluded cases 6

eTable 3-Comparison of demographics, characteristics and outcomes for included and excluded cases for CURB65 6

eTable 5-Comparison of demographics, characteristics and outcomes for included and excluded cases for qSOFA 8

eTable 6-Comparison of demographics, characteristics and outcomes for included and excluded cases for Lac-CURB-65 10

eTable 7-Comparison of demographics, characteristics and outcomes for included and excluded cases for NEWS 12

Missing data analysis- missing data assumed to be normal 14

eTable 8-Performance of CURB65 as a prognostic tool in different outcome measures stratified by CAP aetiology- missing values assumed to be normal 14

eTable 9-Ability of severity assessment tools to risk stratify for different outcome measures in CAP- missing values assumed to be normal 16

eTable 10-Performance characteristics of the severity scoring systems using 30-day mortality as the outcome measure: missing values assumed to be normal 18

Missing data analysis - multiple imputation analysis 19

eTable 11-Performance of CURB65 as a prognostic tool in different outcome measures stratified by CAP aetiology using multiple imputation to account for missing values 19

eTable 12-Ability of severity assessment tools to risk stratify for different outcome measures in CAP using multiple imputation to account for missing values 22

eTable 13-Performance characteristics of the severity scoring systems using 30-day mortality as the outcome measure using multiple imputation 25

eTable 14-Receiver operator characteristic (ROC) curves to assess overall accuracy of severity assessment tools using 30-day mortality as the standard 26

eTable 1-Scoring Parameters for NEWS score

Observation	0	1	2	3
**RR**	12-20	9-11	21-24	<8 or >25
**Oxygen Saturations (%)**	>96	95-94	93-92	<91
**Supplemental oxygen therapy**	No	No	Yes	No
**Temperature (°C)**	36.1-38.0	35.1-36.0 or 38.1-39.0	>39.1	<35.0
**Systolic Blood pressure (mmHg)**	111-219	101-110	91-100	<90 or >219
**Heart rate (BPM)**	51-90	41-50 or 91-110	111-130	<40 or >131
**AVPU score**	Alert			Voice, Pain, Unresponsive

eTable 1 demonstrates the scoring parameters used in the composite NEWS score(1). RR= respiratory rate, BPM= beats per minute, AVPU= alert, voice, pain & unresponsive

eTable 2-Demographics, characteristics and outcomes of study cohort

Characteristic	CAP patients
**N**	**1545**
**Age, median, IQR (years)**	76, 63-85
**Gender n (%)**	
Male	785.0 (50.8)
Female	760.0 (49.2)
**Ethnicity n (%)**	
Caucasian	1302 (84.3)
Indian subcontinent	119 (7.7)
Black*	24 (1.6)
Chinese	3 (0.2)
Other†	49 (3.2)
Unknown‡	48 (3.1)
**Aetiology n (%)**	
HCAP	449 (29.1)
Non-HCAP	1096 (70.9)
**Comorbidity n (%)**	
Cardiovascular disease	827 (53.5)
Chronic pulmonary disease	588 (38.1)
Diabetes mellitus	309 (20.0)
Metabolic disease§	238 (15.4)
Solid tumour malignancy	204 (13.2)
Gastrointestinal disease	181 (11.7)
Rheumatological disease	173 (11.2)
Dementia	170 (11.0)
Psychiatric disease	168 (10.9)
Cerebrovascular disease	161 (10.4)
Haematological disease	119 (7.7)
Neurological disease	119 (7.7)
Other Endocrinopathy	114 (7.4)
Chronic Kidney disease	89 (5.8)
Benign Prostatic Hypertrophy	48 (3.1)
Chronic liver disease	34 (2.2)
Peripheral vascular disease	30 (1.9)
Solid organ or bone marrow transplant	28 (1.8)
Leg ulcers	17 (1.1)
Other//	65 (4.2)
None	134 (11.0)
**Outcomes**	

30 day mortality n (%)	293 (19.0)
90 day mortality n (%)	390 (25.2)
**Characteristic**	**CAP patients**
365 day mortality n (%)	511 (33.1)
In-Hospital death n (%)	238 (15.4)
ICU admission n (%)	99 (6.4)
Hospital readmission within 30 days n (%)	102 (6.6)
Length of in-patient stay, median (IQR) (days)	6.99 (3.65-14.1)

[*] African n=5, Caribbean n=19 [†] Other includes any ethnic group not included in Caucasian, Indian subcontinent, Black, Chinese, Arab or Mixed ethnicity [‡] Data not documented on electronic patient record [§] Includes those with obesity and dyslipidaemia [ll] Includes the following conditions: glaucoma n=11, pressure sore n=6, lymphedema n=4, aortic aneurysm n=3 (thoracic n=1, abdominal n=2), cataracts n=3, macular degeneration n=3, blindness n=2 (unilateral n=1, bilateral n=1), HIV infection n=2, hydroureter n=2, hypoxic brain injury n=2, psoriasis n=2, bullous pemphigoid n=1, cervical spine injury n=1, chronic pain n=1, cystinosis n=1, genital herpes=1, osteomyelitis n=1, previous polio n=1, deafness n=1, Fournier's gangrene n=1, lymph node tuberculosis n=1, obesity-related hypoventilation syndrome n=1, pleural plaques n=1, polycystic ovarian syndrome n=1.
CAP= community acquired pneumonia, HCAP= healthcare associated pneumonia, ICU= intensive care unit.

Comparison of included and excluded cases

Observed characteristics and outcomes for cases included and excluded from analysis due to missing data were compared for each severity scoring system.

eTable 3-Comparison of demographics, characteristics and outcomes for included and excluded cases for CURB65

Characteristic	CURB65	No CURB65*	p value
**N**	**1311**	**234**	
**Age, Median (IQR) (years)**	**77 (63,86)**	**72 (59.75, 84)**	**0.019**
**Gender n (%)**			
Male	665 (50.7)	120 (51.3)	0.875
Female	646 (49.3)	114 (48.7)	
**Ethnicity n (%)**			
Caucasian	1108 (84.5)	194 (82.9)	0.725
Indian subcontinent	98 (7.5)	21 (9.0)	
Black[†]	21 (1.6)	3 (1.3)	
Chinese	3 (0.2)	0 (0.0)	
Other[‡]	43 (3.3)	6 (2.6)	
Unknown[§]	38 (2.9)	10 (4.3)	
**Aetiology n (%)**			
HCAP	375 (28.6)	74 (31.6)	0.349
Non-HCAP	936 (71.4)	160 (69.3)	
**Comorbidity n (%)**			
Cardiovascular disease	692 (52.7)	135 (57.7)	0.166
Chronic pulmonary disease	509 (38.8)	79 (33.8)	0.142
Diabetes mellitus	264 (20.1)	45 (19.2)	0.749
Metabolic disease[ll]	208 (15.9)	30 (12.8)	0.235
Solid tumour malignancy	171 (13.0)	33 (14.1)	0.659
Gastrointestinal disease	158 (12.1)	23 (9.8)	0.330
Rheumatological disease	143 (10.9)	30 (12.8)	0.393
Dementia	156 (11.9)	14 (6.0)	0.01
Psychiatric disease	147 (11.2)	21 (9.0)	0.311
Cerebrovascular disease	142 (10.8)	19 (8.1)	0.211
Haematological disease	87 (6.6)	32 (13.7)	<0.001
Neurological disease	102 (7.8)	17 (7.3)	0.785
Other Endocrinopathy	94 (7.2)	20 (8.5)	0.458
Chronic Kidney disease	63 (4.8)	26 (11.1)	<0.001
Benign Prostatic Hypertrophy	43 (3.3)	5 (2.1)	0.353
Chronic liver disease	24 (1.8)	10 (4.3)	0.02
Peripheral vascular disease	27 (2.1)	3 (1.3)	0.427
Solid organ or bone marrow transplant	13 (1.0)	15 (6.4)	<0.001
Leg ulcers	15 (1.1)	2 (1.0)	0.696
Other[**]	55 (4.2)	10 (4.3)	0.956
None	108 (8.2)	26 (11.1)	0.150
**Outcomes**			
30 day mortality n (%)	260 (19.8)	33 (14.1)	0.039
90 day mortality n (%)	338 (25.8)	52 (22.2)	0.248
365 day mortality n (%)	435 (33.2)	76 (32.5)	0.833
In-Hospital death n (%)	208 (15.9)	30 (12.8)	0.235
ICU admission n (%)	87 (6.6)	12 (5.1)	0.386
Hospital readmission within 30 days n (%)	89 (6.8)	13 (5.6)	0.484
Length of in-patient stay, median (IQR) (days)	7 (4, 15)	7 (4, 7)	0.564

Comparison of proportions performed using Chi square test. Differences in length of in-patient stay compared using the Mann Whitney-U test. *Excluded from the analysis due to missing data on the following parameters: Confusion (230), Urea (5), Respiratory Rate (4), Blood Pressure (0), Age >65 years (0). [†]African n=5, Caribbean n=19 [‡]Other includes any ethnic group not included in Caucasian, Indian subcontinent, Black, Chinese, Arab or Mixed ethnicity [§]Data not documented on electronic patient record [//]Includes those with obesity and dyslipidaemia [e]Includes the following conditions: glaucoma n=11, pressure sore n=6, lymphoedema n=4, aortic aneurysm n=3 (thoracic n=1, abdominal n=2), cataracts n=3, macular degeneration n=3, blindness n=2 (unilateral n=1, bilateral n=1), HIV infection n=2, hydroureter n=2, hypoxic brain injury n=2, psoriasis n=2, bullous pemphigoid n=1, cervical spine injury n=1, chronic pain n=1, cystinosis n=1, genital herpes=1, osteomyelitis n=1, previous polio n=1, deafness n=1, Fournier’s gangrene n=1, lymph node tuberculosis n=1, obesity-related hypoventilation syndrome n=1, pleural plaques n=1, polycystic ovarian syndrome n=1.
HCAP= healthcare associated pneumonia, ICU= intensive care unit

eTable 5-Comparison of demographics, characteristics and outcomes for included and excluded cases for qSOFA

Characteristic	qSOFA	No qSOFA*	p value
**Characteristic**	**1315**	**230**	
**N**	**72 (59, 83)**	**77 (63, 86)**	**0.007**
**Age, Median (IQR) (years)**			
**Gender n (%)**	667 (50.7)	118 (51.3)	0.871
Male	648 (49.3)	112 (48.7)	
Female			
**Ethnicity n (%)**	1111 (84.5)	191 (83.0)	0.816
Caucasian	98 (7.5)	21 (9.1)	
Indian subcontinent	21 (1.6)	3 (1.3)	
Black[†]	3 (0.2)	0 (0.0)	
Chinese	43 (3.3)	6 (2.6)	
Other[‡]	39 (3.0)	9 (3.9)	
Unknown[§]			
**Aetiology n (%)**	376 (28.6)	73 (31.7)	0.332
HCAP	939 (71.4)	157 (68.3)	
Non-HCAP			
**Comorbidity n (%)**	694 (52.8)	133 (57.8)	0.157
Cardiovascular disease	513 (39.0)	75 (32.6)	0.065
Chronic pulmonary disease	266 (20.2)	43 (18.7)	0.592
Diabetes mellitus	208 (15.8)	30 (13.0)	0.282
Metabolic disease[//]	172 (13.1)	32 (13.9)	0.731
Solid tumour malignancy	158 (12.0)	23 (10.0)	0.381
Gastrointestinal disease	143 (10.9)	30 (13.0)	0.336
Rheumatological disease	157 (11.9)	13 (5.7)	0.005
Dementia	147 (11.2)	21 (9.1)	0.357
Psychiatric disease	141 (10.7)	20 (8.7)	0.353
Cerebrovascular disease	87 (6.6)	32 (13.9)	<0.001
Haematological disease	103 (7.8)	16 (7.0)	0.646
Neurological disease	94 (7.1)	20 (8.7)	0.408
Other Endocrinopathy	63 (4.8)	26 (11.3)	<0.001
Chronic Kidney disease	43 (3.3)	5 (2.2)	0.377
Benign Prostatic Hypertrophy	24 (1.8)	10 (4.3)	0.016
Chronic liver disease	27 (2.1)	3 (1.3)	0.448
Peripheral vascular disease	13 (1.0)	15 (6.5)	<0.001
Solid organ or bone marrow transplant	15 (1.1)	2 (0.9)	0.716
Leg ulcers	56 (4.3)	9 (3.9)	0.810
Other[**]	108 (8.2)	26 (11.3)	0.124
None	263 (20.0)	30 (13.0)	0.013
**Outcomes**			
30 day mortality n (%)	341 (25.9)	49 (21.3)	0.136
90 day mortality n (%)	438 (33.3)	73 (31.7)	0.641
365 day mortality n (%)	210 (16.0)	28 (12.2)	0.141
In-Hospital death n (%)	87 (6.6)	12 (5.2)	0.424
ICU admission n (%)	89 (6.8)	13 (5.7)	0.530
Hospital readmission within 30 days n (%)	7 (4, 14)	7 (4, 15)	0.481

Comparison of proportions performed using Chi square test. Differences in length of in-patient stay compared using the Mann Whitney-U test. *Excluded from the analysis due to missing data on the following parameters: Mentation (230), Respiratory Rate (4), and Blood pressure (4).[†]African n=5, Caribbean n=19 [‡]Other includes any ethnic group not included in Caucasian, Indian subcontinent, Black, Chinese, Arab or Mixed ethnicity [§]Data not documented on electronic patient record [//]Includes those with obesity and dyslipidaemia [e]Includes the following conditions: glaucoma n=11, pressure sore n=6, lymphoedema n=4, aortic aneurysm n=3 (thoracic n=1, abdominal n=2), cataracts n=3, macular degeneration n=3, blindness n=2 (unilateral n=1, bilateral n=1), HIV infection n=2, hydroureter n=2, hypoxic brain injury n=2, psoriasis n=2, bullous pemphigoid n=1, cervical spine injury

n=1, chronic pain n=1, cystinosis n=1, genital herpes=1, osteomyelitis n=1, previous polio n=1, deafness n=1, Fournier's gangrene n=1, lymph node tuberculosis n=1, obesity-related hypoventilation syndrome n=1, pleural plaques n=1, polycystic ovarian syndrome n=1. qSOFA= quick Sepsis Related Organ Failure Assessment, HCAP= healthcare associated pneumonia, ICU= intensive care unit

eTable 6-Comparison of demographics, characteristics and outcomes for included and excluded cases for Lac-CURB-65

Characteristic	Lac-CURB-65	No Lac-CURB-65*	p value
**Characteristic**	**1213**	**332**	
**N**	77 (64, 86)	74 (59, 84)	0.008
**Age, Median (IQR) (years)**			
**Gender n (%)**	621 (51.2)	164 (49.4)	0.562
Male	592 (48.8)	168 (50.6)	
Female			
**Ethnicity n (%)**	1029 (84.8)	273 (82.2)	0.695
Caucasian	87 (7.2)	32 (9.6)	
Indian subcontinent	18 (1.5)	6 (1.8)	
Black[†]	2 (0.2)	1 (0.3)	
Chinese	40 (3.3)	9 (2.7)	
Other[‡]	37 (3.1)	11 (3.3)	
Unknown[§]			
**Aetiology n (%)**	342 (28.2)	107 (32.2)	0.151
HCAP	871 (71.8)	225 (67.8)	
Non-HCAP			
**Comorbidity n (%)**	645 (53.2)	182 (54.8)	0.594
Cardiovascular disease	480 (39.6)	108 (32.5)	0.019
Chronic pulmonary disease	244 (20.1)	65 (19.6)	0.828
Diabetes mellitus	190 (15.7)	48 (14.5)	0.590
Metabolic disease[//]	160 (13.2)	44 (13.3)	0.976
Solid tumour malignancy	149 (12.3)	32 (9.6)	0.184
Gastrointestinal disease	127 (10.5)	46 (13.9)	0.083
Rheumatological disease	139 (11.5)	31 (9.3)	0.274
Dementia	141 (11.6)	27 (8.1)	0.070
Psychiatric disease	136 (11.2)	25 (7.5)	0.052
Cerebrovascular disease	73 (6.0)	46 (13.9)	<0.001
Haematological disease	96 (7.9)	23 (6.9)	0.550
Neurological disease	88 (7.3)	26 (7.8)	0.722
Other Endocrinopathy	52 (4.3)	37 (11.1)	<0.001
Chronic Kidney disease	37 (3.1)	11 (3.3)	0.807
Benign Prostatic Hypertrophy	22 (1.8)	12 (3.6)	0.048
Chronic liver disease	26 (2.1)	4 (1.2)	0.272
Peripheral vascular disease	9 (0.7)	19 (5.7)	<0.001
Solid organ or bone marrow transplant	14 (1.2)	3 (0.9)	0.698
Leg ulcers	53 (4.4)	12 (3.6)	0.544
Other[**]	100 (8.2)	34 (10.2)	0.252
None	252 (20.8)	41 (12.3)	0.001
**Outcomes**			
30 day mortality n (%)	321 (26.5)	69 (20.8)	0.035
90 day mortality n (%)	415 (34.2)	96 (28.9)	0.069
365 day mortality n (%)	201 (16.6)	37 (11.1)	0.015
In-Hospital death n (%)	57 (7.2)	12 (3.6)	0.019
ICU admission n (%)	78 (6.4)	24 (7.2)	0.604
Hospital readmission within 30 days n (%)	7 (4, 14)	7 (3, 14.75)	0.260

Comparison of proportions performed using Chi square test. Differences in length of in-patient stay compared using the Mann Whitney-U test. *Excluded from the analysis due to missing data on the following parameters: Confusion (230), Urea (5), Respiratory Rate (4), Blood Pressure (0), Age >65 years (0), lactate (227) [†]African n=5, Caribbean n=19 [‡]Other includes any ethnic group not included in Caucasian, Indian subcontinent, Black, Chinese, Arab or Mixed ethnicity [§]Data not documented on

electronic patient record [d]Includes those with obesity and dyslipidaemia [e]Includes the following conditions: glaucoma n=11, pressure sore n=6, lymphoedema n=4, aortic aneurysm n=3 (thoracic n=1, abdominal n=2), cataracts n=3, macular degeneration n=3, blindness n=2 (unilateral n=1, bilateral n=1), HIV infection n=2, hydroureter n=2, hypoxic brain injury n=2, psoriasis n=2, bullous pemphigoid n=1, cervical spine injury n=1, chronic pain n=1, cystinosis n=1, genital herpes=1, osteomyelitis n=1, previous polio n=1, deafness n=1, Fournier's gangrene n=1, lymph node tuberculosis n=1, obesity-related hypoventilation syndrome n=1, pleural plaques n=1, polycystic ovarian syndrome n=1.HCAP= healthcare associated pneumonia, ICU= intensive care unit

eTable 7-Comparison of demographics, characteristics and outcomes for included and excluded cases for NEWS

Characteristic	NEWS	No NEWS*	p value
**Characteristic**	**1534**	**11**	
**N**	76 (63, 85)	79 (42, 83)	0.450
**Age, Median (IQR) (years)**			
**Gender n (%)**	782 (51.0)	3 (27.3)	0.117
Male	752 (49.0)	8 (72.7)	
Female			
**Ethnicity n (%)**	1292 (84.2)	10 (90.9)	0.750
Caucasian	119 (7.8)	0 (0.0)	
Indian subcontinent	24 (1.6)	0 (0.0)	
Black[†]	3 (0.2)	0 (0.0)	
Chinese	48 (3.1)	1 (9.1)	
Other[‡]	48 (3.1)	0 (0.0)	
Unknown[§]			
**Aetiology n (%)**	449 (29.3)	0 (0.0)	0.033
HCAP	1085 (70.9)	11 (100.0)	
Non-HCAP			
**Comorbidity n (%)**	824 (53.7)	3 (27.3)	0.080
Cardiovascular disease	584 (38.1)	4 (36.4)	0.908
Chronic pulmonary disease	308 (20.1)	1 (9.1)	0.364
Diabetes mellitus	236 (15.4)	2 (18.2)	0.798
Metabolic disease[//]	202 (13.2)	2 (18.2)	0.625
Solid tumour malignancy	180 (11.7)	1 (9.1)	0.786
Gastrointestinal disease	172 (11.2)	1 (9.1)	0.824
Rheumatological disease	170 (11.1)	0 (0.0)	0.242
Dementia	167 (10.9)	1 (9.1)	0.849
Psychiatric disease	160 (10.4)	1 (9.1)	0.885
Cerebrovascular disease	119 (7.8)	0 (0.0)	0.336
Haematological disease	118 (7.7)	1 (9.1)	0.862
Neurological disease	112 (7.3)	2 (18.2)	0.169
Other Endocrinopathy	89 (5.8)	0 (0.0)	0.411
Chronic Kidney disease	48 (3.1)	0 (0.0)	0.551
Benign Prostatic Hypertrophy	33 (2.2)	1 (9.1)	0.118
Chronic liver disease	30 (2.0)	0 (0.0)	0.640
Peripheral vascular disease	26 (1.7)	2 (18.2)	<0.001
Solid organ or bone marrow transplant	17 (1.1)	0 (0.0)	0.726
Leg ulcers	65 (4.2)	0 (0.0)	0.485
Other[**]	133 (8.7)	1 (9.1)	0.961
None	290 (18.9)	3 (27.3)	0.481
**Outcomes**			
30 day mortality n (%)	386 (25.2)	4 (36.4)	0.394
90 day mortality n (%)	505 (32.9)	6 (54.5)	0.129
365 day mortality n (%)	235 (15.3)	3 (27.3)	0.274
In-Hospital death n (%)	95 (6.2)	4 (36.4)	<0.001
ICU admission n (%)	102 (6.6)	0 (0.0)	0.376
Hospital readmission within 30 days n (%)	7 (4, 14)	7 (2, 13)	0.994

Comparison of proportions performed using Chi square test. Differences in length of in-patient stay compared using the Mann Whitney-U test. *Excluded from the analysis due to missing data on the following parameters: Temperature (9), Oxygen Saturations (5), Level of consciousness (4), Respiratory Rate (4), Blood pressure (4), Heart rate (4) [†]African n=5, Caribbean n=19 [‡]Other includes any ethnic group not included in Caucasian, Indian subcontinent, Black, Chinese, Arab or Mixed ethnicity [§]Data not documented on electronic patient record [//]Includes those with obesity and dyslipidaemia [e]Includes the following conditions: glaucoma n=11, pressure sore n=6, lymphoedema n=4, aortic aneurysm n=3 (thoracic n=1, abdominal n=2),

cataracts n=3, macular degeneration n=3, blindness n=2 (unilateral n=1, bilateral n=1), HIV infection n=2, hydroureter n=2, hypoxic brain injury n=2, psoriasis n=2, bullous pemphigoid n=1, cervical spine injury n=1, chronic pain n=1, cystinosis n=1, genital herpes=1, osteomyelitis n=1, previous polio n=1, deafness n=1, Fournier's gangrene n=1, lymph node tuberculosis n=1, obesity-related hypoventilation syndrome n=1, pleural plaques n=1, polycystic ovarian syndrome n=1.NEWS= National Early Warning Score, HCAP= healthcare associated pneumonia, ICU= intensive care unit

Missing data analysis- missing data assumed to be normal

To account for missing data we repeated the analyses with all missing values assumed to be normal, this data is presented below.

eTable 8-Performance of CURB65 as a prognostic tool in different outcome measures stratified by CAP aetiology- missing values assumed to be normal

**Outcome**	**CURB65 Score**						**p value**
	**0**	**1**	**2**	**3**	**4**	**5**	
**n**							
All	213	370	477	337	130	18	-
HCAP	34	89	140	122	54	10	-
Non-HCAP	179	281	337	215	76	8	-
**30 day mortality n (%)**							
All	6.0 (2.8)	39.0 (10.5)	91.0 (19.1)	91.0 (27.0)	59.0 (45.4)	7.0 (38.9)	<0.001
HCAP	0.0 (0.0)	12.0 (13.5)	34.0 (24.3)	39.0 (32.0)	27.0 (50.0)	3.0 (30.0)	<0.001
Non-HCAP	6.0 (3.4)	27.0 (9.6)	57.0 (16.9)	25.0 (24.2)	32.0 (42.1)	4.0 (50.0)	<0.001
**90 day mortality n (%)**							
All	12.0 (5.6)	56.0 (15.1)	132.0 (27.7)	117.0 (34.7)	66.0 (50.8)	7.0 (38.9)	<0.001
HCAP	1.0 (2.9)	17.0 (19.1)	40.0 (28.6)	50.0 (41.0)	29.0 (53.7)	3.0 (30.0)	<0.001
Non-HCAP	11.0 (6.1)	39.0 (13.9)	92.0 (27.3)	67.0 (31.2)	37.0 (48.7)	4.0 (50.0)	<0.001
**365 day mortality n (%)**							
All	14.0 (6.6)	87.0 (23.5)	174.0 (36.5)	155.0 (46.0)	74.0 (56.9)	7.0 (38.9)	<0.001
HCAP	1.0 (2.9)	33.0 (37.1)	59.0 (42.1)	60.0 (49.2)	34.0 (63.0)	3.0 (30.0)	<0.001

Non-HCAP	13.0 (7.3)	54.0 (19.2)	115.0 (34.1)	99.0 (44.2)	40.0 (52.6)	4.0 (50.0)	<0.001
**In-Hospital death n (%)**							
All	3.0 (1.4)	30.0 (8.1)	76.0 (15.9)	75.0 (22.3)	49.0 (37.7)	5.0 (27.8)	<0.001
HCAP	0.0 (0.0)	10.0 (11.2)	24.0 (17.1)	28.0 (23.0)	19.0 (35.2)	1.0 (10.0)	<0.001
Non-HCAP	3.0 (1.7)	20.0 (7.1)	52.0 (15.4)	47.0 (21.9)	30.0 (39.5)	4.0 (50.0)	<0.001
**ICU admission n (%)**							
All	13.0 (6.1)	19.0 (5.1)	43.0 (9.0)	16.0 (4.7)	6.0 (4.7)	2.0 (11.1)	0.883
HCAP	2.0 (5.9)	4.0 (4.5)	5.0 (3.6)	4.0 (3.3)	1.0 (1.9)	1.0 (10.0)	0.545
Non-HCAP	11.0 (6.1)	15.0 (5.3)	38.0 (11.3)	12.0 (5.6)	5.0 (6.6)	1.0 (12.5)	0.525
**Length of in-patient stay median days, (IQR)**							
All	3.0 (1.0, 7.0)	6.0 (3.0, 13.0)	8.0 (4.0, 16.0)	9.0 (5.0, 17.0)	8.0 (4.0 – 13.3)	8.5 (4.5, 14.0)	<0.001
HCAP	5.0 (3.0, 10.0)	9.0 (4.0, 16.0)	10.0 (6.0, 16.8)	8.0 (4.8, 16.0)	8.0 (5.0, 12.0)	7.5 (4.5, 17.3)	0.575
Non-HCAP	3.0 (1.0, 7.0)	6.0 (3.0, 11.5)	8.0 (4.0, 15.5))	10.0 (5.0, 20.0)	7.0 (4.0, 16.0)	9.0 (3.8, 12.3)	<0.001

Missing data assumed to have normal values. Comparison of proportions performed using Chi square test for trend, trends in median length of stay assessed using the Jonckheere-Terpstra test. ICU= intensive care unit, NEWS= National Early Warning Score, SIRS= Systemic Inflammatory Response Syndrome criteria, qSOFA= quick Sepsis Related Organ Failure Assessment, IQR= interquartile range

eTable 9-Ability of severity assessment tools to risk stratify for different outcome measures in CAP- missing values assumed to be normal

Outcome	CURB65						p value
	0<br>n=213	1<br>n=370	2<br>n=477	3<br>n=337	4<br>n=130	5<br>n=18	
30 day mortality n (%)	6.0 (2.8)	39.0 (10.5)	91.0 (19.1)	91.0 (27.0)	59.0 (45.4)	7.0 (38.9)	<0.001
90 day mortality n (%)	12.0 (5.6)	56.0 (15.1)	132.0 (27.7)	117.0 (34.7)	66.0 (50.8)	7.0 (38.9)	<0.001
365 day mortality n (%)	14.0 (6.6)	87.0 (23.5)	174.0 (36.5)	155.0 (46.0)	74.0 (56.9)	7.0 (38.9)	<0.001
In-Hospital death n (%)	3.0 (1.4)	30.0 (8.1)	76.0 (15.9)	75.0 (22.3)	49.0 (37.7)	5.0 (27.8)	<0.001
ICU admission n (%)	13.0 (6.1)	19.0 (5.1)	43.0 (9.0)	16.0 (4.7)	6.0 (4.7)	2.0 (11.1)	0.883
Length of in-patient stay median days, (IQR)	3.0 (1.0, 7.0)	6.0 (3.0, 13.0)	8.0 (4.0, 16.0)	9.0 (5.0, 17.0)	8.0 (4.0 – 13.3)	8.5 (4.5, 14.0)	<0.001
	Lac-CURB-65						
	Low<br>n=356		Moderate<br>n=618		High<br>n=571		
30 day mortality n (%)	15.0 (4.2)		93.0 (15.0)		185.0 (32.4)		<0.001
90 day mortality n (%)	33.0 (9.3)		135.0 (21.8)		222.0 (38.9)		<0.001
365 day mortality n (%)	55.0 (15.4)		184.0 (29.8)		272.0 (47.6)		<0.001
In-Hospital death n (%)	12.0 (3.4)		77.0 (12.5)		149.0 (26.1)		<0.001
ICU admission n (%)	15.0 (4.2)		46.0 (7.4)		38.0 (6.7)		0.209
Length of in-patient stay median days, (IQR)	5.0 (2.0, 10.0)		7.0 (4.0, 14.0)		8.0 (5.0, 16.0)		0.005

	NEWS			
	Low<br>n=564	Moderate<br>n=420	High<br>n=561	
30 day mortality n (%)	63.0<br>(11.2)	62.0<br>(14.8)	168.0<br>(29.9)	<0.001
90 day mortality n (%)	98.0<br>(17.4)	95.0<br>(22.6)	197.0<br>(35.1)	<0.001
365 day mortality n (%)	140.0<br>(24.8)	128.0<br>(30.5)	243<br>(43.3)	<0.001
In-Hospital death n (%)	58.0<br>(10.3)	53.0<br>(12.6)	127.0<br>(22.6)	<0.001
ICU admission n (%)	15.0<br>(2.7)	25.0<br>(6.0)	59.0<br>(10.5)	<0.001
Length of in-patient stay median days, (IQR)	6.0<br>(3.0 – 12.0)	7.0<br>(4.0, 16.0)	8.0<br>(4.0, 14.5)	<0.001

	qSOFA				
	0<br>n=539	1<br>n=685	2<br>n=269	3<br>n=52	
30 day mortality n (%)	64.0<br>(11.9)	119.0<br>(17.4)	86.0<br>(32.0)	24.0<br>(46.2)	<0.001
90 day mortality n (%)	94.0<br>(17.4)	167.0<br>(24.4)	104.0<br>(38.7)	25.0<br>(48.1)	<0.001
365 day mortality n (%)	135.0<br>(25.0)	225.0<br>(32.8)	123.0<br>(45.7)	28.0<br>(53.8)	<0.001
In-Hospital death n (%)	56.0<br>(10.4)	91.0<br>(13.3)	71.0<br>(26.4)	20.0<br>(38.5)	<0.001
ICU admission n (%)	21.0<br>(3.9)	52.0<br>(7.6)	23.0<br>(8.6)	3.0<br>(5.8)	0.016
Length of in-patient stay median days, (IQR)	6.0<br>(3.0, 12.0)	8.0<br>(4.0, 15.0)	8.0<br>(4.0, 15.0)	7.5<br>(5.0, 15.8)	<0.001

Missing data assumed to have normal values. Comparison of proportions performed using Chi square test for trend, trends in median length of stay assessed using the Jonckheere-Terpstra test. ICU= intensive care unit, NEWS= National Early Warning Score, qSOFA= quick Sepsis Related Organ Failure Assessment, IQR= inter-quartile range

eTable 10-Performance characteristics of the severity scoring systems using 30-day mortality as the outcome measure: missing values assumed to be normal

Score	Sensitivity (%)	Specificity (%)	PPV (%)	NPV (%)	NLR	PLR
**CURB65 ≥2**<br>962 (62.3%)	84.6	43.0	25.8	92.3	0.36	1.48
**CURB65 ≥3**<br>485 (31.4%)	53.6	73.8	32.4	87.2	0.63	2.05
**Lac-CURB-65 ≥Moderate**<br>1189 (77.0%)	94.9	27.2	23.4	95.8	0.19	1.3
**Lac-CURB-65 High**<br>571 (37.0%)	63.1	69.2	32.4	88.9	0.53	2.05
**qSOFA**<br>321 (20.8%)	37.5	83.15	34.3	85.1	0.75	2.23
**NEWS ≥medium**<br>981 (63.4%)	78.5	40.0	23.5	88.8	0.54	1.31
**NEWS High**<br>561 (36.3%)	57.3	68.6	30.0	87.3	0.62	1.83

30-day mortality as standard. PPV: positive predictive value, NPV: negative predictive value, NLR: negative likelihood ratio, PLR: positive likelihood ratio. Cut off value for both qSOFA and SIRS was ≥2. NEWS= National Early Warning Score, SIRS= Systemic Inflammatory Response Syndrome criteria, qSOFA= quick Sequential (Sepsis-related) Organ Failure Assessment.

Missing data analysis - multiple imputation analysis

To further address the issue of missing data, we have performed a multiple imputation analysis.

We performed five imputations, the combined results for the different analyses can be seen below. Included in the model were all parameters with missing data. In addition, factors that were found to be significantly different between cases with a complete data set for any particular severity scoring system and those without were included in the model (See eTables 4-7). These included aetiology of CAP (HCAP/non-HCAP), history of haematological diagnosis, chronic kidney disease, solid organ transplant, chronic liver disease, chronic lung disease and dementia. The results presented are of the pooled analysis from the five different imputations with the range of p values.

eTable 11-Performance of CURB65 as a prognostic tool in different outcome measures stratified by CAP aetiology using multiple imputation to account for missing values

**Outcome**	**CURB65 Score**						**p value range**
	**0**	**1**	**2**	**3**	**4**	**5**	
**n**							
**All**	206	352.6	467	356.6	144.2	18.6	
**HCAP**	33.2	80.8	134	131.6	59.4	10	
**Non-HCAP**	172.8	271.8	333	225	84.8	8.6	
**30 day mortality n (%)**							
All	6.0 (2.9)	36.0 (10.2)	85.4 (18.3	95.8 (26.9)	62.2 (43.1)	7.6 (40.9)	<0.001*
HCAP	0.0 (0.0)	10.6 (13.1)	31.6 (23.6)	41.6 (31.6)	28.2 (47.5)	3.0 (30.0)	<0.001*

Non-HCAP	6.0 (3.5)	25.4 (9.3)	53.8 (16.2)	54.2 (24.1)	34.0 (40.1)	4.6 (53.5)	<0.001*
**90 day mortality n (%)**							
All	11.4 (5.5)	51.8 (14.7)	122.8 (26.3)	126.2 (35.4)	70.2 (48.7)	7.6 (40.9)	<0.001*
HCAP	1.0 (3.0)	15.6 (19.3)	35.8 (26.7)	53.4 (40.6)	31.2 (52.5)	3.0 (30.0)	<0.001*
Non-HCAP	10.4 (6.0)	36.2 (13.3)	87.0 (26.1)	72.8 (32.4)	39.0 (46.0)	4.6 (53.5)	<0.001*
**365 day mortality n (%)**							**p-value**
All	13.4 (6.5)	79.8 (22.6)	162.8 (34.9)	167.1 (47.1)	79.6 (55.2)	7.6 (40.9)	<0.001*
HCAP	1.0 (3.0)	30.0 (37.1)	54.6 (40.7)	63.8 (48.5)	37.6 (63.3)	3.0 (30.0)	<0.001*
Non-HCAP	12.4 (7.2)	49.8 (18.3)	108.2 (32.5)	104.0 (46.2)	42.0 (49.5)	4.6 (53.5)	<0.001*
**In-Hospital death n (%)**							
All	3.0 (1.5)	27.6 (7.8)	71.2 (15.2)	78.4 (22.0)	52.2 (36.2)	5.6 (30.1)	<0.001*
HCAP	0.0 (0.0)	8.6 (10.6)	22.0 (16.4)	30.2 (22.9)	20.2 (34.0)	1.0 (1.0)	<0.001*
Non-HCAP	3.0 (1.7)	19.0 (7.0)	49.2 (14.8)	48.2 (21.4)	32.0 (37.7)	4.6 (53.5)	<0.001**

**ICU admission n (%)**							
All	12.4 (6.0)	17.2 (4.9)	43.2 (9.3)	17.0 (4.8)	7.2 (5.0)	2.0 (10.8)	0.724 – 0.973
HCAP	2.0 (6.0)	3.4 (4.2)	5.2 (3.9)	3.4 (2.6)	2.0 (3.4)	1.0 (10.0)	0.550 – 0.818
Non-HCAP	10.4 (6.0)	13.8 (5.1)	38.0 (11.4)	13.6 (6.0)	5.2 (6.1)	1.0 (11.6)	0.378 – 0.677
**Length of in-patient stay median days, (IQR)**							
All	3.0 (1.0, 7.0)	6.0 (3.0, 13.0)	8.0 (4.0, 16.0)	9.0 (5.0, 18.0)	8.0 (4.0, 14.3)	8.0 (4.0, 14.0)	<0.001*
HCAP	5.0 (3.0, 10.0)	8.5 (4.0, 16.5)	10.0 (5.0, 17.5)	8.0 (5.0, 16.0)	8.0 (5.0, 13.0)	7.5 (4.5, 17.3)	0.170 – 0.500
Non-HCAP	3.0 (1.0, 7.0)	6.0 (3.0, 12.0)	8.0 (4.0, 16.0)	9.0 (4.0, 16.0)	8.0 (4.0, 17.5)	8.0 (3.5, 12.3)	<0.001*

Comparison of proportions performed using Chi square test for trend, trends in median length of stay assessed using the Jonckheere-Terpstra test. *All p-values <0.001. ICU= intensive care unit, NEWS= National Early Warning Score, SIRS= Systemic Inflammatory Response Syndrome criteria, qSOFA= quick Sequential (Sepsis-related) Organ Failure Assessment, IQR= inter-quartile range

eTable 12-Ability of severity assessment tools to risk stratify for different outcome measures in CAP using multiple imputation to account for missing values

Outcome	CURB65						p value range
	**0 n=206**	**1 n=352.6**	**2 n=467**	**3 n=356.6**	**4 n=144.2**	**5 n=18.6**	
30 day mortality n (%)	6.0 (2.9)	36.0 (10.2)	85.4 (18.3	95.8 (26.9)	62.2 (43.1)	7.6 (40.9)	<0.001*
90 day mortality n (%)	11.4 (5.5)	51.8 (14.7)	122.8 (26.3)	126.2 (35.4)	70.2 (48.7)	7.6 (40.9)	<0.001*
365 day mortality n (%)	13.4 (6.5)	79.8 (22.6)	162.8 (34.9)	167.1 (47.1)	79.6 (55.2)	7.6 (40.9)	<0.001*
In-Hospital death n (%)	3.0 (1.5)	27.6 (7.8)	71.2 (15.2)	78.4 (22.0)	52.2 (36.2)	5.6 (30.1)	<0.001*
ICU admission n (%)	12.4 (6.0)	17.2 (4.9)	43.2 (9.3)	17.0 (4.8)	7.2 (5.0)	2.0 (10.8)	0.724 – 0.973
Length of in-patient stay median days, (IQR)	3.0 (1.0, 7.0)	6.0 (3.0, 13.0)	8.0 (4.0, 16.0)	9.0 (5.0, 18.0)	8.0 (4.0, 14.3)	8.0 (4.0, 14.0)	<0.001*
	**Lac-CURB-65**						
	**Low n=275.2**		**Moderate n=638.2**		**High n=631.6**		
30 day mortality n (%)	10.4 (3.8)		88.6 (13.9)		194.0 (30.7)		<0.001*
90 day mortality n (%)	21.0 (7.6)		131.2 (20.6)		237.8 (37.7)		<0.001*
365 day mortality n (%)	38.2 (13.9)		179.8 (28.2)		293.0 (46.4)		<0.001*

In-Hospital death n (%)	7.0 (2.5)	73.4 (11.5)	157.6 (25.0)	<0.001*
ICU admission n (%)	11.2 (4.1)	46.0 (7.8)	41.8 (6.6)	0.090 – 0.499
Length of in-patient stay median days, (IQR)	5.0 (2.0, 10.0)	8.0 (3.0, 14.0)	8.5 (5.0, 16.0)	<0.001*
	**NEWS**			
	**Low n=564**	**Moderate n=420**	**High n=561**	
30 day mortality n (%)	61.8 (11.0)	61.4 (14.7)	169.8 (30.0)	<0.001*
90 day mortality n (%)	96.8 (17.2)	94.4 (22.6)	198.8 (35.2)	<0.001*
365 day mortality n (%)	138.8 (24.7)	126.8 (30.3)	254.4 (43.4)	<0.001*
In-Hospital death n (%)	56.8 (10.1)	52.4 (12.5)	128.8 (22.8)	<0.001*
ICU admission n (%)	13.8 (2.5)	25.4 (6.1)	59.8 (10.6)	<0.001*
Length of in-patient stay median days, (IQR)	6.0 (3.0, 12.0)	7.0 (4.0, 16.0)	8.0 (4.0, 14.0)	<0.001*

	**qSOFA**				
	**0 n=485.4**	**1 n=716.6**	**2 n=289.8**	**3 n= 53.2**	
30 day mortality n (%)	54.0 (11.1)	127.0 (17.7)	86.8 (30.0)	25.2 (47.4)	<0.001*
90 day mortality n (%)	78.4 (16.2)	177.4 (24.8)	108.0 (37.3)	26.2 (49.2)	<0.001*

365 day mortality n (%)	113.2 (23.3)	239.0 (33.4)	129.6 (44.7)	29.2 (54.9)	<0.001*
In-Hospital death n (%)	46.2 (9.5)	99.4 (13.9)	71.4 (24.6)	21.0 (39.5)	<0.001*
ICU admission n (%)	16.0 (3.3)	55.6 (7.8)	24.2 (8.4)	3.2 (6.0	0.003 – 0.025
Length of in-patient stay median days, (IQR)	6.0 (2.0, 12.0)	7.0 (4.0, 15.0)	8.0 (4.0, 16.0)	8.0 (4.8, 16.0)	<0.001*

Comparison of proportions performed using Chi square test for trend, trends in median length of stay assessed using the Jonckheere-Terpstra test. *All p-values <0.001ICU= intensive care unit, NEWS= National Early Warning Score, qSOFA= quick Sequential (Sepsis-related) Organ Failure Assessment tool, IQR= inter-quartile range

eTable 13-Performance characteristics of the severity scoring systems using 30-day mortality as the outcome measure using multiple imputation

Score	Sensitivity (%)	Specificity (%)	PPV (%)	NPV (%)	NLR	PLR
**CURB65 ≥2** 986 (63.8%)	85.7	41.3	25.5	92.5	0.35	1.46
**CURB65 ≥3** 519 (33.6%)	56.7	71.7	31.9	87.6	0.60	2.00
**Lac-CURB-65 ≥Moderate** 1270 (82.2%)	94.9	27.2	23.4	95.8	0.19	1.3
**Lac-CURB-65 High** 632 (40.9%)	63.1	69.2	32.4	88.9	0.53	2.05
**qSOFA** 343 (22.2%)	38.2	81.55	32.7	84.9	0.76	2.07
**NEWS ≥medium** 983 (63.6%)	78.8	39.9	23.5	89.0	0.53	1.31
**NEWS High** 565 (36.6%)	58.0	68.5	30.1	87.5	0.61	1.84

Analysis performed using pooled values from multiple imputation n=1545. PPV: positive predictive value, NPV: negative predictive value, NLR: negative likelihood ratio, PLR: positive likelihood ratio. Cut off value for qSOFA was ≥2. NEWS= National Early Warning Score, qSOFA= quick Sequential (Sepsis-related) Organ Failure Assessment.

eTable 14-Receiver operator characteristic (ROC) curves to assess overall accuracy of severity assessment tools using 30-day mortality as the standard

Imputation Model	Severity assessment tool	AUROC
1	CURB65	0.70
	Lac-CURB-65	0.68
	NEWS	0.64
	qSOFA	0.63
2	CURB65	0.70
	Lac-CURB-65	0.68
	NEWS	0.64
	qSOFA	0.63
3	CURB65	0.70
	Lac-CURB-65	0.68
	NEWS	0.64
	qSOFA	0.64
4	CURB65	0.70
	Lac-CURB-65	0.68
	NEWS	0.65
	qSOFA	0.63
5	CURB65	0.70
	Lac-CURB-65	0.68
	NEWS	0.64
	qSOFA	0.63

AUROC= area under receiver operator curve, NEWS= National Early Warning Score, qSOFA= quick Sequential (Sepsis-related) Organ Failure Assessment.

Bibliography

1. Royal College of Physicans. National Early Warning Score(NEWS) 2 Standardising the assessment of acute-illness severity in the NHS. 2017.
